# Machine vision-based autonomous road hazard avoidance system for self-driving vehicles

**DOI:** 10.1038/s41598-024-62629-4

**Published:** 2024-05-28

**Authors:** Chengqun Qiu, Hao Tang, Yuchen Yang, Xinshan Wan, Xixi Xu, Shengqiang Lin, Ziheng Lin, Mingyu Meng, Changli Zha

**Affiliations:** 1 School of Automotive Engineering, Yancheng Institute of Technology, Yancheng, 224051 China; 2 Jiangsu Province Intelligent Optoelectronic Devices and Measurement-Control Engineering Research Center, Yancheng Teachers University, Yancheng, 224007 China; 3 School of Automotive and Transportation Engineering, Hefei University of Technology, Anhui, 230009 China; 4 Interdisciplinary Graduate School of Science & Engineering, Tokyo Institute of Technology, Yokohama, 2268502 Japan; 5 School of Electronic Engineering and Intelligent Manufacturing, Anqing Normal University, Anhui, 246133 China

**Keywords:** Deep learning, Machine vision, Self-Driving, Risk avoidance, YOLOv5s, Control algorithm, Mechanical engineering, Computer science

## Abstract

The resolution of traffic congestion and personal safety issues holds paramount importance for human’s life. The ability of an autonomous driving system to navigate complex road conditions is crucial. Deep learning has greatly facilitated machine vision perception in autonomous driving. Aiming at the problem of small target detection in traditional YOLOv5s, this paper proposes an optimized target detection algorithm. The C3 module on the algorithm’s backbone is upgraded to the CBAMC3 module, introducing a novel GELU activation function and EfficiCIoU loss function, which accelerate convergence on position loss *l*_*box*_, confidence loss *l*_*obj*_, and classification loss *l*_*cls*_, enhance image learning capabilities and address the issue of inaccurate detection of small targets by improving the algorithm. Testing with a vehicle-mounted camera on a predefined route effectively identifies road vehicles and analyzes depth position information. The avoidance model, combined with Pure Pursuit and MPC control algorithms, exhibits more stable variations in vehicle speed, front-wheel steering angle, lateral acceleration, etc., compared to the non-optimized version. The robustness of the driving system's visual avoidance functionality is enhanced, further ameliorating congestion issues and ensuring personal safety.

## Introduction

Deep learning has made remarkable strides in the field of autonomous driving and Advanced Driver Assistance Systems^1,2^. However, the driving safety of such systems has not gained widespread societal acceptance. As a result, research and validation of autonomous driving in complex road conditions continue unabated. It is worth noting that environmental perception in autonomous driving systems heavily relies on deep learning technology^3^. Prior to the application of deep learning in machine vision, visual perception technology was largely stagnant^4,5^. Images captured by onboard cameras are critical for intelligent perception in autonomous driving systems.

This paper aims to optimize the YOLOv5s object detection model and employ an optimized camera visual ranging strategy to address the challenges mentioned above. In conjunction with the optimized visual model, a local obstacle avoidance approach is adopted. Additionally, the paper combines the Pure Pursuit algorithm^6,7^ and the Model Predictive Control (MPC) algorithm^8,9^ to evaluate the collision avoidance functionality of the experimental autonomous vehicle. The chosen flat terrain in Jiangsu, known for its well-developed manufacturing and commercial centers, is ideal for testing autonomous driving technology. These areas frequently face traffic congestion issues. The approach is designed to tackle complex road conditions, adverse weather scenarios, and improve the accuracy and robustness of visual algorithms for target identification and tracking in complex road environments. Three innovative optimizations ve been applied to enhance the visual model:

- (1) EfficiCIoU Loss Function: A new EfficiCIoU loss function is introduced, addressing the limitations of the traditional CIoU function. The traditional CIoU function involves numerous tricks to handle the IoU of predicted and anchor boxes, which increases computational complexity and does not consider the IoU for small targets, leading to sample imbalance issues. The new EfficiCIoU loss function improves the IoU, incorporates a context mechanism to better understand environmental information, and enhances the detection of small or densely packed objects;
- (2) Integration of CBAM Attention Mechanism and YOLOv5s C3 Module: The paper integrates the CBAM attention mechanism with the YOLOv5s C3 module. The new CBAMC3 module combines channel attention and spatial attention, enhancing the model’s focus on input features. It is embedded within the CNN framework, improving the inference speed of the visual model;
- (3) Upgrade to GELU Activation Function: The traditional ReLU activation function is upgraded to the GELU activation function. Compared to the traditional linear activation function, GELU is more conducive to feature mapping in models because it does not exhibit saturation in input values, thus addressing the gradient vanishing problem.

## Methods

### Machine vision convolutional neural networks

Deep learning frameworks are well-suited for representation learning^10–12^, employing multi-layered nonlinear transformations^13,14^ in an efficient manner. Due to their cost-effectiveness, high precision, and robustness, they find extensive applications in the field of autonomous driving. In convolutional neural network (CNN)^15–17^, the convolutional kernels extract features while reducing the parameter count. Pooling layers shrink the size of feature maps while retaining essential information. Fully connected layers typically operate at the top of the network, mapping convolutional kernels and feature maps to output categories, connecting one or more hidden layers to the output layer. Activation functions work between the convolutional and fully connected layers, enabling the model to handle more complex tasks. Different activation functions serve various purposes in processing the output layer's tasks. The multi-layered architecture described above facilitates feature extraction by the model.

**Figure 1:**
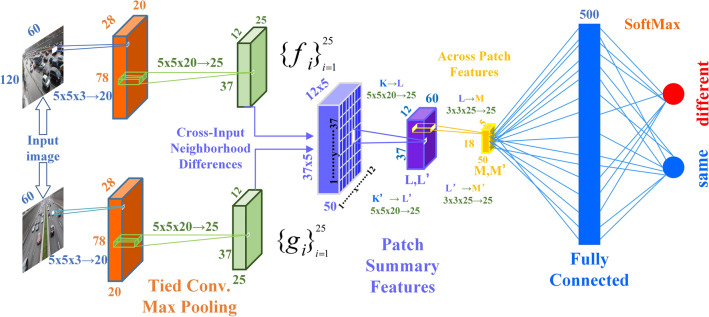
Structure diagram of a deep learning-based machine vision convolutional neural network nested layer by layer.

Figure 1 illustrates the architecture of a deep learning network for image processing. It starts with a Convolution (Conv) layer^18^, which extracts useful features from input images using 5 × 5 filters with a depth of 3 (corresponding to RGB color channels). This generates 20 different feature maps, highlighting specific features from the input. Next is the "Tied Conv Max Pooling" layer^19,20^, where “Tied Conv” means parameter sharing, reducing the model's size and preventing overfitting. "Max Pooling" downsizes feature maps by selecting maximum values in different regions, preserving essential features. The "Cross-Input Neighborhood Differences" step enhances matching by computing local differences between input images, useful for stereo vision matching tasks. “Patch Summary Features” are compact features from small patches, often generated by aggregating local features. “In the Across Patch Features” step, features from multiple image blocks are compared and integrated to create a global feature representation, aiding understanding of the entire image scene. Fully connected layers flatten output feature maps into one-dimensional vectors, which enter a network for classification. Finally, the SoftMax layer^21^ transforms fully connected layer output into a probability distribution, crucial for classifying images in the vehicle vision system. Neural networks, with their multi-layered architecture and advanced feature extraction capabilities, enable thorough analysis of visual data from vehicles, providing essential environmental perception capabilities for autonomous driving systems.

### YOLOv5s object detection modeling

**Figure 2:**
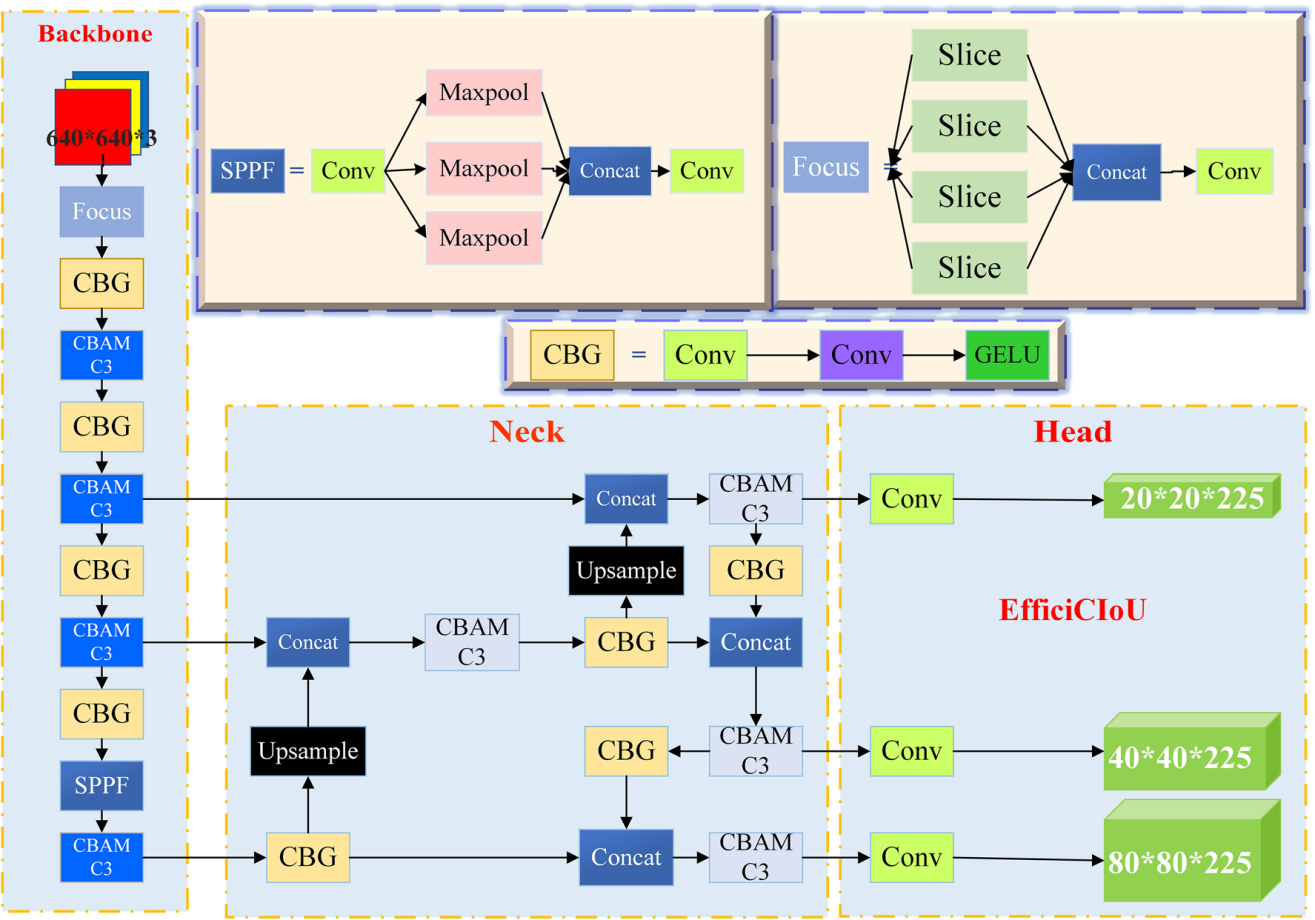
The basic framework of the improved YOLOv5s algorithm.

Machine vision has become a crucial research area in the field of autonomous driving. In object detection algorithms, the YOLOv5s algorithm utilizes convolutional neural networks to calculate the positions of objects to be recognized^22–24^, classifying and localizing them accurately. YOLOv5s is a high-accuracy neural network that surpasses the limitations of traditional image processing algorithms. It is a one-stage algorithm known for its fast inference speed, with frame rates suitable for autonomous driving systems. As decipted in Fig. 2, the network's input comprises 640 × 640 three-channel images, typically divided into grid regions of 80 × 80, 40 × 40, and 20 × 20. The network's output includes predictions for all grid regions, including classification probabilities, confidence scores, and bounding box information for objects in those regions.

Key enhancements to the YOLOv5s algorithm include data augmentation using Mosaic^25,26^, adaptive anchor box calculations, and adaptive image scaling. The backbone employs operations such as convolution and pooling to reduce feature map dimensions, increase depth, and incorporate the CBAM C3 module for automatic attention to image features. Feature fusion (Neck) is achieved using a PAN + FPN^27^ structure, merging feature maps with different resolutions and rich semantic information, creating a feature pyramid.

The convolution layers in the backbone network and feature fusion section (Neck) use the GELU activation function to introduce non-linearity, facilitating the capture of complex image features. The final detection head (Head) is responsible for outputting object detection results, including bounding boxes, confidence scores, and class information. During model training, the EfficiCIoU LOSS function continuously calculates gradients and updates parameters to achieve convergence. The YOLOv5s model is iteratively trained to obtain a set of weight parameters that minimize the loss function^28^. The depth of grid regions in the network relates to their accuracy, with shallower grids providing higher accuracy, particularly for detecting small objects. Careful grid processing is also necessary to avoid semantic ambiguity.

The FPN + PAN structure combines feature maps of different scales in a hierarchical manner, similar to the SSD approach^29^, enabling layer-wise processing and strong feature fusion capabilities. This structure is a crucial module in hierarchical detection methods and facilitates further feature fusion. The vehicle is equipped with a camera that captures RGB images, which are fed into the YOLOv5s algorithm. In the Backbone phase, image preprocessing is performed, including normalization to match the input size of the network. The system employs adaptive anchor box calculations and image scaling, while Mosaic data augmentation is used during grid training to improve model accuracy and speed. In addition to the CBAMC3 module, the Backbone also uses the Focus architecture as a base for general feature extraction. The GELU activation function after each convolutional layer aids in capturing complex image features^30^. The Neck network sits between the base network and the detection head, enhancing feature diversity and robustness. The Head is responsible for outputting the results of object detection, and the number of branches depends on the specific detection method, often used for classification and data regression.

### Camera adaptation processing

The system utilizes a stereo camera setup, and prior to conducting experiments, the camera system undergoes a series of processing steps^31,32^. Initially, the stereo cameras perform real-time image capture, capturing full stereo images. Subsequently, stereo matching algorithms are applied to these images to calculate disparities, thereby enabling depth estimation. Deep learning algorithms are then employed for object recognition within the images. Based on the identified object classes and their respective spatial extents, distance and orientation information for the objects is extracted from the disparity map. With this information in hand, a well-designed obstacle avoidance strategy is devised to control the vehicle's actions. This strategy includes actions such as emergency braking or steering adjustments to facilitate automatic obstacle avoidance, ensuring safe vehicle operation. The overarching goal is to enable the vehicle to navigate safely in its environment by leveraging the stereo camera system for distance and object recognition.

**Figure 3:**
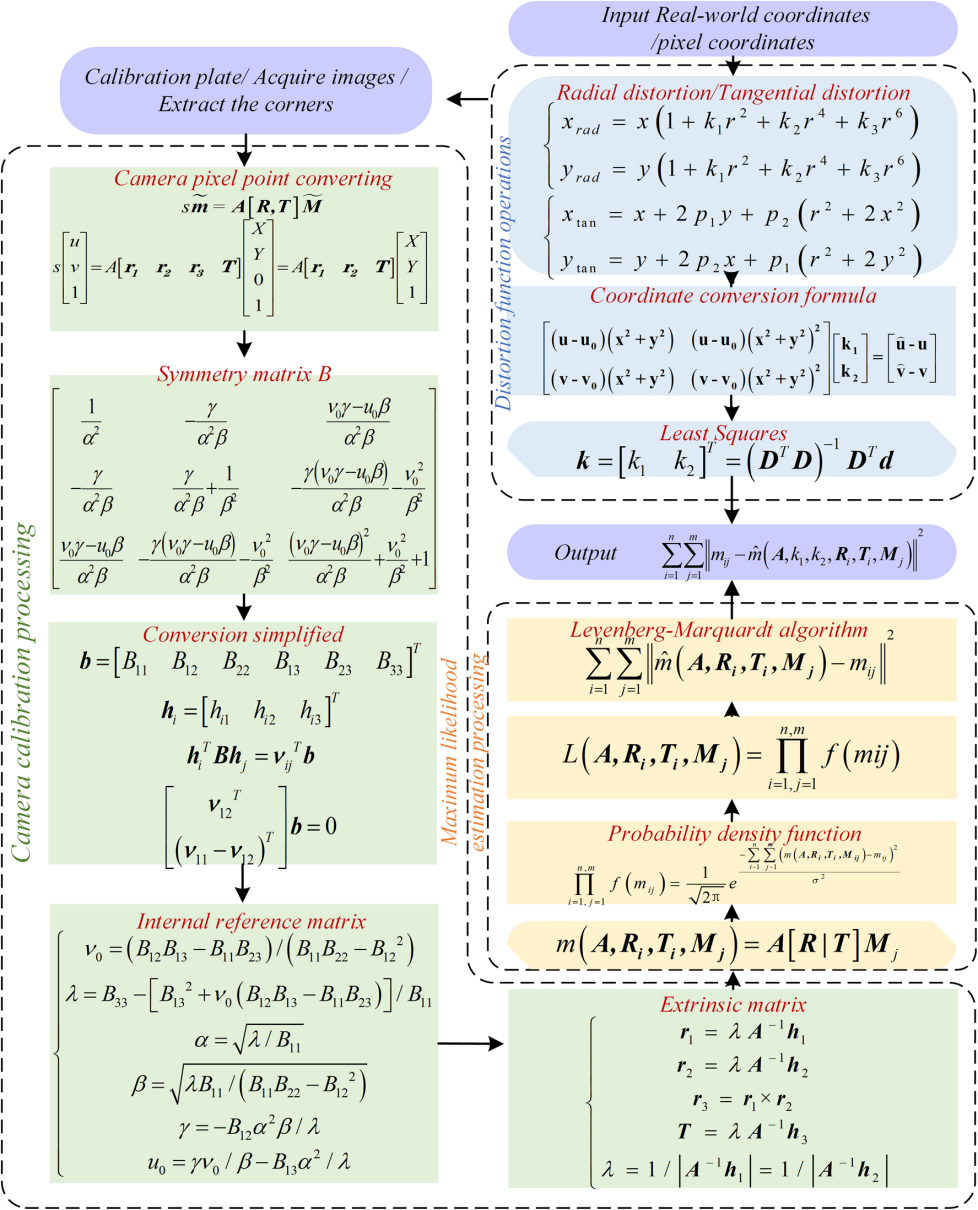
Algorithm flow of internal and external parameters and distortion coefficients of the camera.

Given the world coordinates of a real point M = [X,Y,Z]^T^ and its corresponding camera pixel coordinates m = [u, v]^T^, as shown in Fig. 3, the transformation from M to m, combined with a scale factor s, the vertical coordinate of the calibration board in the real world Z_W_ = 0, and simplification using the symmetric matrix **B**, is used to determine the camera's internal parameters by capturing images of a chessboard pattern. Once the camera's intrinsic parameter matrix is computed, the extrinsic parameter matrix can be obtained.

During the process of solving both intrinsic and extrinsic parameters, factors such as lens distortion, image resolution, and noise levels can affect the estimation of these parameters. Therefore, it is necessary to introduce the maximum likelihood estimation to optimize the parameters.

Now, combining with the camera parameters in Table 1, for each chessboard image containing m corner points, their corresponding image projection points can be represented as:

1 $$m\left( {\user2{A,R}_{{\varvec{i}}} \user2{,T}_{{\varvec{i}}} \user2{,M}_{{\varvec{j}}} } \right) = {\varvec{A}}\left[ {\user2{R|T}} \right]{\varvec{M}}_{j}$$

**Table 1:** Camera parameters.

Camera	Specifications	Parameters
	Output resolution	2 × (2208 × 1242) @15fps
		2 × (1920 × 1080) @30fps
		2 × (1280 × 720) @60fps
		2 × (672 × 376) @100fps
	Sensor type	1/3·4MP CMOS
Stereo Camera	Baseline	120 mm
	Focal Length	2.12 mm
	Visual Angle	Max.110°(H) × 70°(V) × 120°(D)
	Pixel size	2 μm × 2 μm

Subsequently, the probability density function and likelihood function for the chessboard corner points are derived. The Levenberg–Marquardt algorithm (L-M algorithm) is employed to maximize the likelihood function. The L-M algorithm^33^ introduces a control parameter λ, which is used to balance the gradient descent and Gauss–Newton steps. In each iteration, λ value is dynamically adjusted based on the current parameter estimate and information from the Hessian matrix to balance the two steps, thereby facilitating a faster convergence towards the optimal solution.

2 $$\sum\limits_{i = 1}^{n} {\sum\limits_{j = 1}^{m} {\left\| {\hat{m}\left( {\user2{A,R}_{{\varvec{i}}} \user2{,T}_{{\varvec{i}}} \user2{,M}_{{\varvec{j}}} } \right) - m_{ij} } \right\|} }^{2}$$

Combining the pixel coordinates under the ideal and actual models, camera optical point coordinates, continuous image coordinates under undistorted and radial distortion conditions, the radial distortion parameters are calculated using the least squares method. By incorporating the undistorted intrinsic and extrinsic parameters with the radial distortion parameter k, the estimation of all parameters is achieved, resulting in:

3 $$\sum\limits_{i = 1}^{n} {\sum\limits_{j = 1}^{m} {\left\| {m_{ij} - \hat{m}\left( {{\varvec{A}},k_{1} ,k_{2} ,{\varvec{R}}_{i} ,{\varvec{T}}_{i} ,{\varvec{M}}_{j} } \right)} \right\|}^{2} }$$

After completing camera calibration and obtaining the optimal intrinsic and extrinsic parameter matrices along with the distortion coefficients considering radial distortion, the notation m(***A****,k*_*1*_*,k*_*2*_*,****R***_***i****,*_***T***_***i***_*,****M***_***j***_) represents the coordinates corresponding to the *j* point on the *i* image, accounting for radial distortion.

## Experiments and analysis

### Object detection algorithm optimization

In the calculation of the loss function in the head Section^34^, we introduce the theoretically superior EfficiCIoU_Loss, which improves the components of position loss *l*_*box*_, confidence loss *l*_*obj*_, and classification loss *l*_*cls*_.

Calculation of position loss *l*_*box*_:

4 $$l_{box} = 1 - \frac{{I{\text{o}}U - enclose\_area}}{IoU + \varepsilon }$$

Calculation of confidence loss *l*_*obj*_:

5 $$\begin{gathered} l_{obj} = \sum\limits_{i = 0}^{{s^{2} }} {\sum\limits_{j = 0}^{B} {I_{ij}^{obj} \left\{ {\widehat{{C_{i} }}\log \left( {Ci} \right) + \left( {1 - \widehat{{C_{i} }}} \right)\log \left( {1 - \widehat{{C_{i} }}} \right)} \right\}} } - \hfill \\ \lambda_{noobj} \sum\limits_{i = 0}^{{s^{2} }} {\sum\limits_{j = 0}^{B} {I_{ij}^{noobj} \left\{ {\log \left( {C_{i} } \right) + \left( {1 - \widehat{{C_{i} }}} \right) + \left( {1 - \widehat{{C_{i} }}} \right)\log \left( {1 - \widehat{{C_{i} }}} \right)} \right\}} } \hfill \\ \end{gathered}$$

Calculation of the confidence of classified losses *l*_*cls*_:

6 $$l_{cls} = \sum\limits_{i = 0}^{{s^{2} }} {l_{ij}^{obj} \sum\nolimits_{c \in classes} {\left\{ {\widehat{{P_{i} }}\left( c \right)\log \left( {p_{i} \left( c \right)} \right) + 1 - \widehat{{P_{i} }}\left( c \right)\log \left( {p_{i} \left( c \right)} \right)} \right\}} }$$

EfficiCIoU_Loss combines the concepts from EfficientDet and CIoU, assuming the presence of two bounding boxes: the predicted box and the target box. EfficiCIoU_Loss comprises the following components:

7 $$Localization\_error = \left( {x_{p} - x_{t} } \right)^{2} + \left( {y_{p} - y_{t} } \right)^{2}$$

8 $$Size\_error = \left( {w_{p} - w_{t} } \right)^{2} + \left( {h_{p} - h_{t} } \right)^{2}$$

9 $$CIoU\_error = 1 - IoU + \frac{\alpha v}{{1 - IoU + v}}$$

EfficiCIoU_Loss computes the final loss value as a combination of these error terms, and the specific form may vary depending on different implementations. The design objective of this loss function is to better handle the position and size of the target boxes, ultimately improving object detection performance. Different implementations of YOLOv5s may have varying parameter settings to adapt to different tasks and datasets, allowing for flexibility and customization to suit specific requirements.

#### Experimental setup

The training of the YOLOv5s algorithm model in this system was conducted on a Windows operating system using the Pytorch framework. The system ran on CUDA 11.0 and Python 3.9, and the hardware configuration of the workstation included an Intel Xeon Gold 6248R CPU and a GeForce RTX 4090 GPU. The dataset used for the model is a self-made dataset of 5607 photos from Car recorder and KITTI, an Open Access dataset for autonomous driving training. The dataset is roughly divided into a training set of 70%, a validation set of 15%, and a test set of 15%. The model is trained with 70% of the images from the self-made dataset, as the self-made dataset is collected for specific driving environments or scenarios, and the model can be better adapted to these specific conditions by using the self-made dataset for training. training can make the model better adapted to these specific conditions. The remaining 30% are randomly selected from KITTI, the Open Access dataset that allows the model to be trained in more driving scenarios. For model validation and testing, more than 90% of the images are from KITTI, and the diversity of samples for validation and testing allows the model to be evaluated under a wider range of driving conditions that have not been seen before, and helps to assess the model's generalization ability. Cross-use of self-made datasets and Open Access datasets balances the performance and applicability of the model and enhances the reliability of the model.

The object detection system divided the collected images from vehicles on the road into multiple objects, annotated the dataset with labels, and then split it into training and validation sets. The model underwent multiple rounds of training where parameters were continually updated through gradient descent. During training, minor changes in model training parameters were amplified with the increase in the number of layers. Furthermore, changes in parameters at different layers altered the data distribution in those layers, posing significant challenges to model training.

Deep learning networks have achieved significant success in large-scale image and video recognition, thanks to the development of large public image databases like ImageNet and high-performance computing systems. Through deep learning, the decision-making process in autonomous driving is achieved in an end-to-end manner, directly mapping sensor-collected image information to vehicle driving behavior without extensive manual feature engineering.

Prof. Karim and others^35^ introduced a transformation from existing univariate time series classification models to multivariate time series classification models by incorporating LSTM-FCN. They enhanced the fully convolutional block with squeeze and excitation blocks to further improve accuracy. This framework can be used to build an end-to-end CNN-based steering controller for vehicles, predicting the future distribution of vehicle motion based on current camera observations and vehicle states, and predicting the required steering wheel angle from continuous video images.

Scholars like Li^36^ proposed a novel Simultaneous Localization and Mapping (SLAM) method, namely Attention-SLAM, which combines a visual saliency model with traditional monocular visual SLAM. This approach mimics human navigation patterns, and the generated saliency map can focus more on the same salient object.

**Figure 4:**
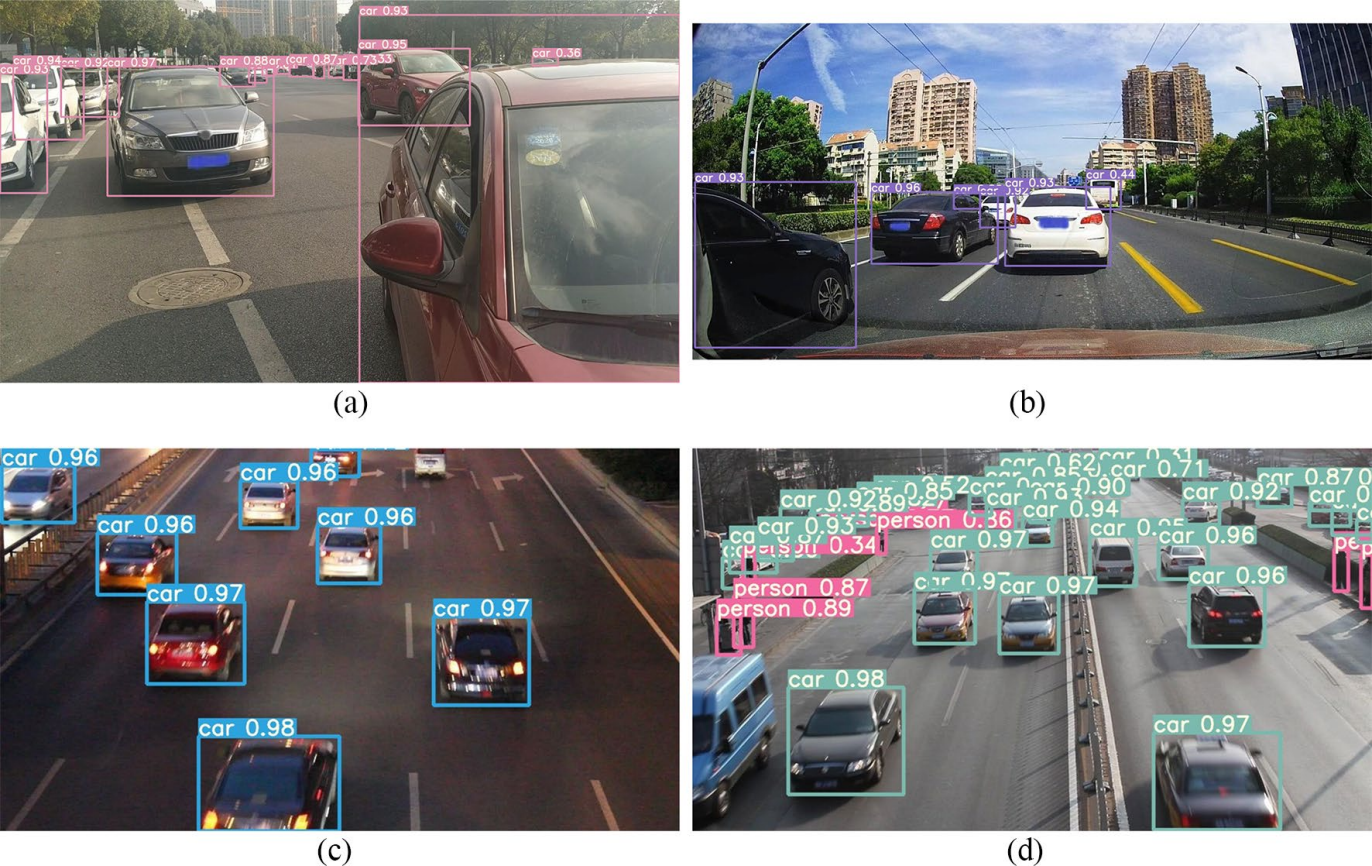
YOLOv5s targets detect road vehicles and pedestrians.

Drawing from the experiences of previous researchers, we further optimized visual perception under the deep learning framework. The pre-trained YOLOv5s model was deployed on the vehicle's onboard camera for inference. In Fig. 4(**a,b,c,d**), even in scenarios with multiple objects within the camera's field of view, distant or relatively small and blurry objects, the inference process exhibited strong accuracy. The confidence level of the algorithm's object inference capability remained around 0.95. This makes it highly suitable for application in scenarios involving multiple lanes, multi-vehicle tracking, or traffic congestion.

#### Optimize results and analysis

We conducted a comprehensive optimization of the traditional YOLOv5s architecture to enhance the accuracy of small object detection and the overall performance of the system. Initially, within the backbone network, we replaced the original C3 module with the CBAMC3 module, which integrates the CBAM^37,38^ attention mechanism. This modification significantly bolstered the network's ability to extract features in complex scenes, particularly enhancing sensitivity and recognition precision in detecting small objects. In the neck and head networks, we substituted the original activation method with the GELU^39^ activation function. Owing to its unique non-linear characteristics, the GELU function improved the feature transmission and transformation processes, rendering the model more effective and precise in handling targets of various sizes. We implemented the EfficiCIoU loss function in the head network, a novel loss function that optimizes the model's convergence behavior during training, especially in the precise computation of localization loss *l*_*box*_, confidence loss *l*_*obj*_, and classification loss *l*_*cls*_. EfficiCIoU significantly heightens localization accuracy by more accurately measuring the similarity between predicted and actual bounding boxes, thereby further enhancing the model's ability to detect small objects. Through these targeted improvements, our model demonstrated superior performance in practical applications, particularly in the identification of road vehicles and the analysis of in-depth positional information within autonomous driving systems.

**Figure 5:**
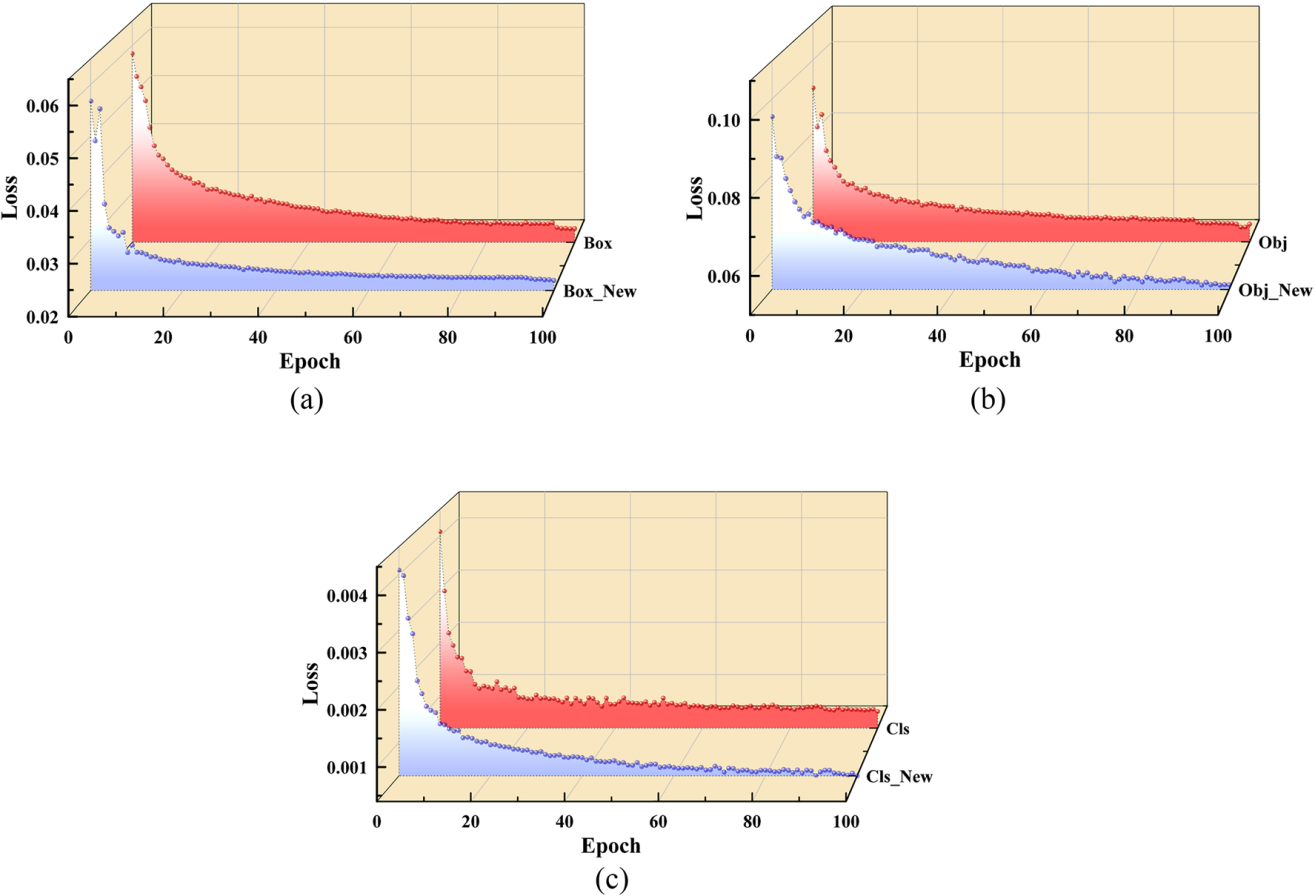
(**a**) Position loss convergence change; (**b**) Convergence change of confidence loss; (**c**) Convergence change of classification loss.

**Figures 6:**
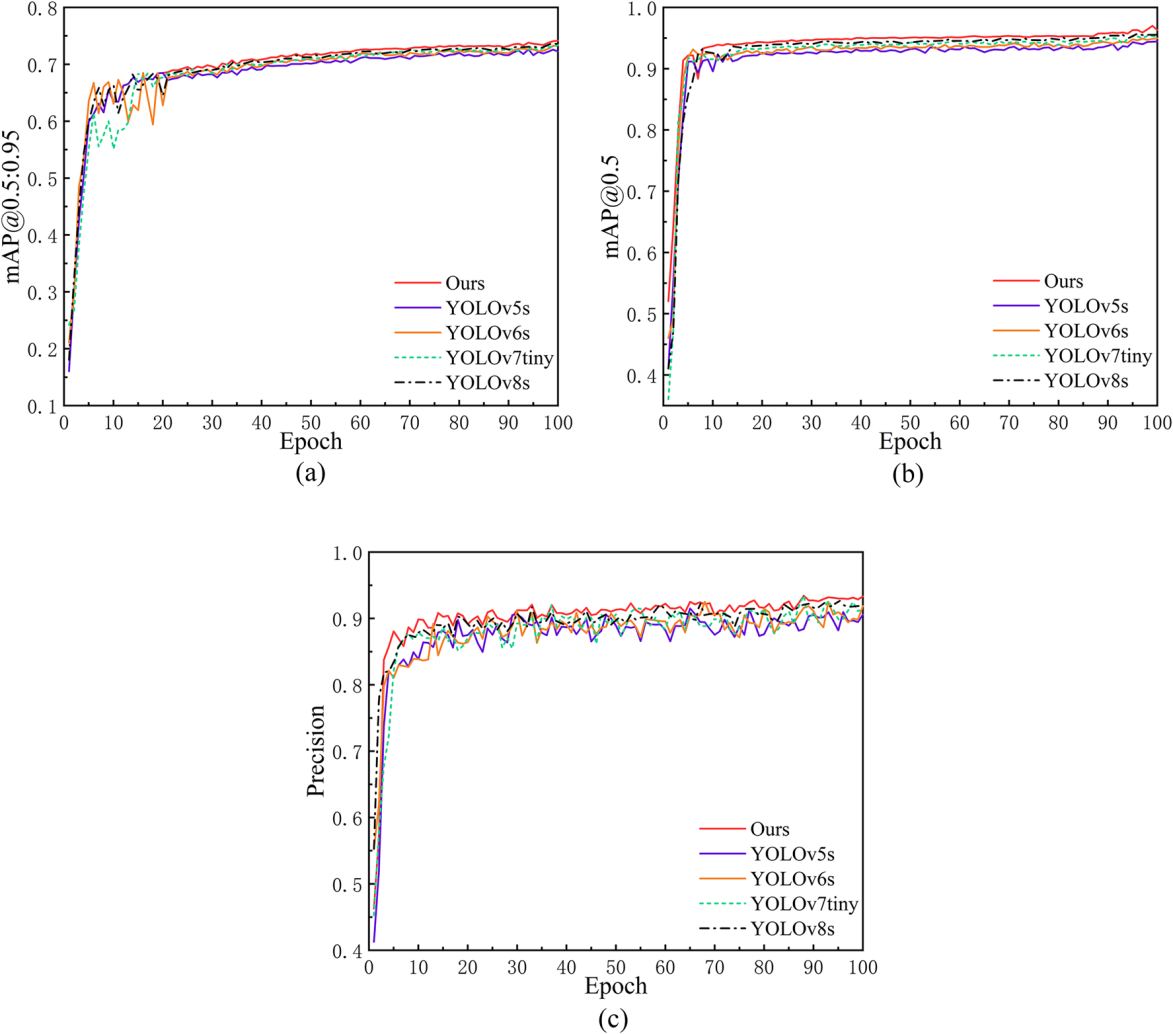
A Comparative analysis of our model with other leading object detection models.

Since the model's loss function tends to decrease with training iterations, we have selected data from the first 100 training rounds. Please refer to Fig. 5 (a,b,c)for the convergence speed changes in the three loss types for training dataset samples and Fig. 6 for the comparative analysis of our model with other leading object detection models. In comparison to the original YOLOv5s model, our modified architecture exhibits enhancements of 1.9%, 2.1%, and 2.73% in mAP@0.5:0.95, mAP@0.5, and Precision, respectively. Against the state-of-the-art YOLOv8, our model demonstrates marginal improvements of 0.4%, 0.64%, and 1.33% across these metrics.

#### Ablation experiments

Ablation experiments are a common method used in deep learning research to evaluate the specific contribution of certain parts of the model to the final performance by removing or replacing them. In the YOLOv5s vehicle detection task, ablation experiments help us clarify which features are critical for improving detection accuracy, accelerating inference, and optimizing model size. In this way, we can gain a deeper understanding of how the model works and optimize it effectively in real-world applications. From Table 2, we can find that model optimization improves the accuracy, recall and mAP of the model to a certain extent, and the model also achieves precise control over the computational resource requirements. Although Params and GFLOPs were increased, they did not put significant pressure on storage resources. In addition, the moderate increase in GFLOPs indicates that the increase in required computation is limited, suggesting that the model can maintain efficient operation even in systems with limited computational resources. The slight improvement in inference Time, which is still maintained in the millisecond range, ensures the model's real-time responsiveness in fast dynamic environments. These improvements highlight the utility of our approach, making it a strong candidate for resource-constrained self-driving vehicle deployments.

**Table 2:** Results of the ablation study of the optimized model in our dataset.

CBAM		GELU		EfficiCIoU	P	R	mAP@0.5	mAP@0.5:0.95		FPS	Params	GFLOPs	Time
					0.891	0.833	0.941		0.725	66	7.02	16.01	0.0091
**√**					0.910	0.890	0.949		0.729	52	11.32	19.94	0.0107
	**√**				0.899	0.857	0.943		0.721	67	8.33	13.71	0.0100
			**√**		0.899	0.855	0.950		0.727	58	8.45	14.10	0.0123
**√**	**√**				0.919	0.891	0.956		0.732	48	11.76	20.00	0.0128
**√**			**√**		0.920	0.893	0.943		0.737	48	12.00	20.92	0.0147
	**√**		**√**		0.904	0.889	0.947		0.730	54	9.11	15.88	0.0111
**√**	**√**		**√**		0.933	0.896	0.963		0.740	47	12.01	21.69	0.0150

#### Comparison of algorithm complexity

In order to measure the computational complexity of the algorithms, we evaluate the number of parameters, the amount of operations, and the inference time of each algorithm, which will provide us with a more accurate performance comparison of our model. These experiments not only validate the practicality of our model, but also provide reliable data support for future optimization and application. In the following table, it can be seen that our algorithm is only 0.32 M higher than YOLOv8s in terms of the parameters, but the Precision and mAP values have been almost similar to YOLOv8s performance, and the GFLOPs of our algorithm has been significantly reduced by 24.91% from 28.90 to 21.69 in YOLOv8s. It signifies that our algorithm improves computational efficiency while maintaining efficient functionality. The inference time is 0.015 s, which is 8.59% faster compared to 0.0163 s in YOLOv8s. This speedup not only implies faster processing, but also reflects the fact that our model is able to provide more efficient performance in real-world applications, especially when it comes to real-time object detection in driving vehicles (Table 3).

**Table 3:** Algorithm Complexity Comparison.

Algorithm	P	R	mAP@0.5	mAP@0.5:0.95	Params	GFLOPs	Time
YOLOv5s	0.891	0.833	0.941	0.725	7.02	16.01	0.0091
YOLOv6s	0.910	0.854	0.949	0.733	16.32	37.94	0.0114
YOLOv7tiny	0.915	0.857	0.949	0.729	6.33	13.71	0.0115
YOLOv8s	0.928	0.885	0.960	0.741	11.69	28.90	0.0163
Ours	0.933	0.896	0.963	0.740	12.01	21.69	0.0150

### Camera vision model testing and analysis

An optimization model equipped with a Stereo Camera is deployed to achieve real-time tracking and target detection functionality for autonomous vehicles. The optimization algorithm is utilized to obtain spatial positioning information of target vehicles. Testing is conducted on selected road segments within the Jiangsu Yancheng High-Tech Industrial Development Zone. This zone encompasses well-developed manufacturing and commercial centers, which often face traffic congestion issues and carry a certain risk of traffic accidents. Therefore, researching an avoidance system for autonomous driving is particularly suitable in this context. The vehicle-mounted camera detects target frames, providing coordinate information of the target vehicle and the distance from the detected vehicle to the center of the vehicle-mounted camera.

**Figures 7:**
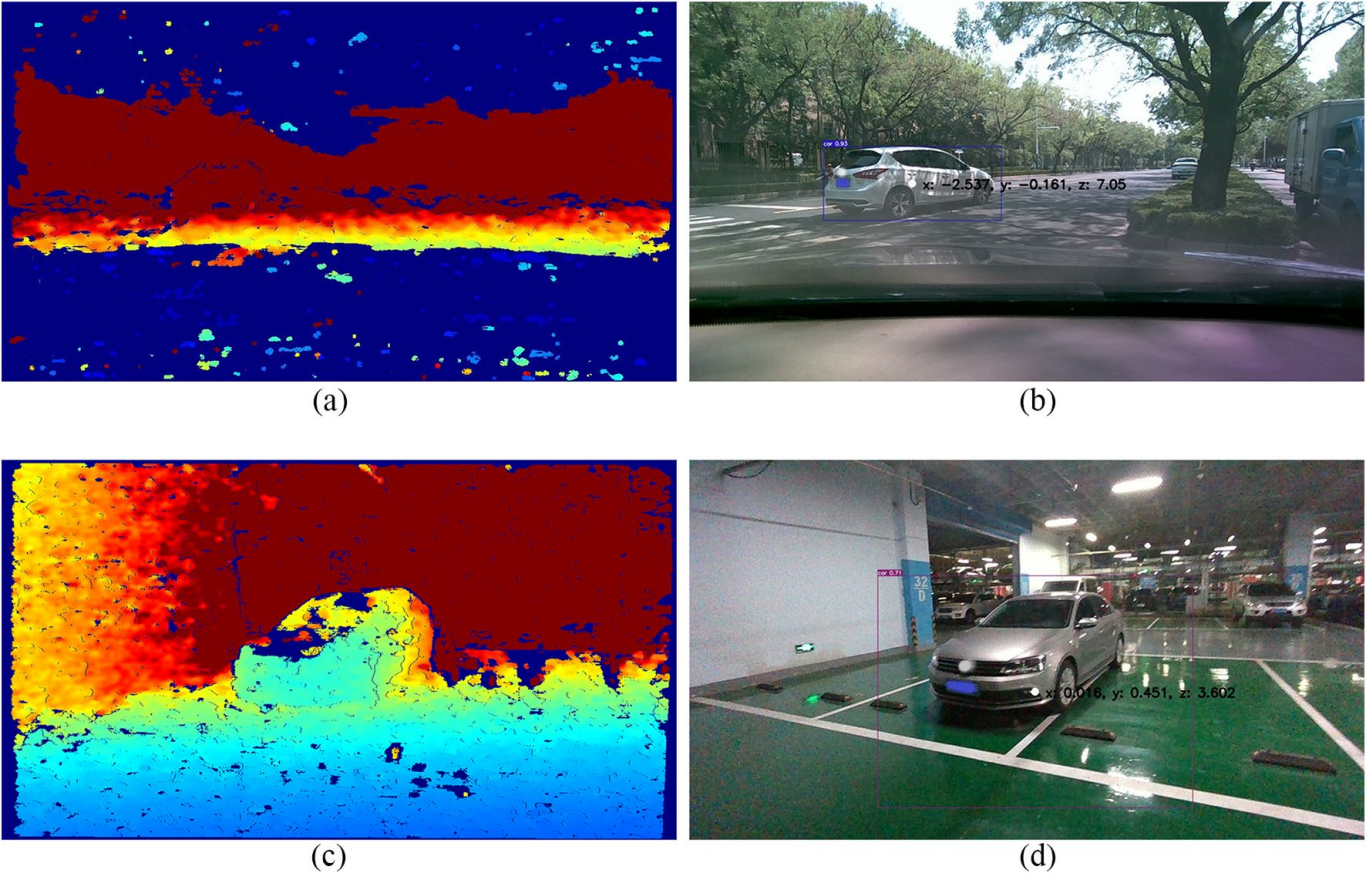
(**a**) The target's depth information; (**b**) Location information of the vehicle while it is moving; (**c**) The target's depth information; (**d**) Location information for parked vehicles.

By combining deformable convolution modules with depth information selection modules, the YOLOv5s model integrated with the accompanying camera can perform tasks related to target identification, tracking, and provide feedback on the depth information of target positions. As shown in Fig. 7(a–d), the on-road performance of the detection model is depicted: (b) illustrates a scenario with a road target vehicle located at coordinates (X: − 2.537, Y: − 0.161) in the two-dimensional coordinate system of the vehicle's field of view, at a distance of 7.05 m from the host vehicle. (d) presents a scenario where the target vehicle is parked at coordinates (X: 0.016, Y: 0.451) in the two-dimensional coordinate system of the vehicle's field of view, at a distance of 3.602 m from the host vehicle.

### Modeling and analysis of hazard avoidance vehicles

To ensure the experimental feasibility rigorously, this simulation adopts a combined simulation approach, integrating Matlab/Simulink, Carsim, and Prescan. Carsim is used to configure the vehicle's dynamic model, Prescan provides the simulated environmental scenarios, and data processing is performed in Matlab/Simulink^40,41^. The algorithms employed include Pure Pursuit and MPC control for local planning and tracking control, respectively.

Obstacle vehicles are placed on the road, categorized as stationary vehicles and moving vehicles, simulating scenarios where the vehicle encounters both stationary obstacles and vehicles in motion while driving on the road. This comprehensive approach ensures a thorough evaluation of the autonomous driving system's performance in various real-world situations.

#### 50 km/h experiment

A simulation scenario was established using Carsim to simulate obstacle avoidance for autonomous vehicles within a predefined environment. In this integrated simulation, the autonomous vehicle was configured to cruise at a speed of 50 km/h while navigating through a simulated scenario that included both static obstacles and moving vehicles on the road. The purpose of this joint simulation was to assess whether the vehicle could still operate effectively under the new system. This comprehensive simulation test aimed to evaluate whether the vehicle could continue to function efficiently and safely, including the ability to avoid obstacles, within this altered operational framework.

**Figure 8:**
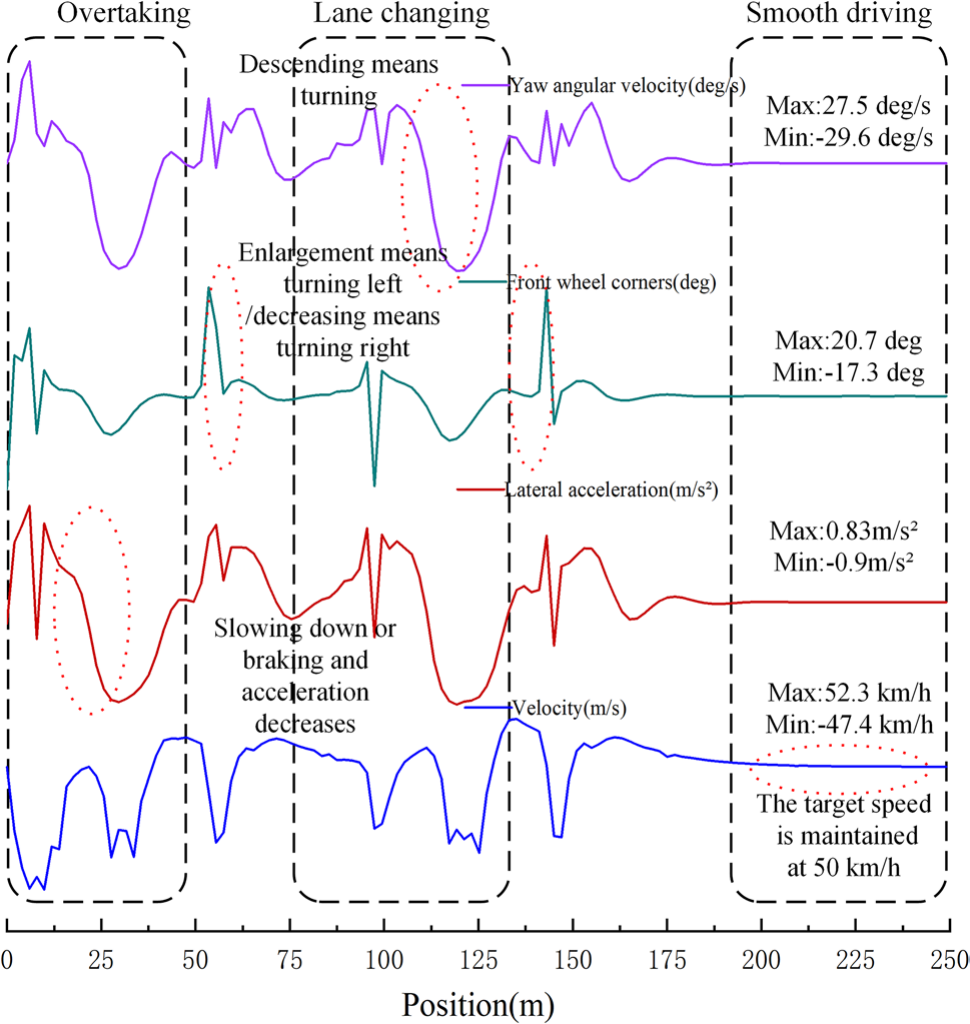
Speed, lateral acceleration, front wheel rotation angle, and yaw angular velocity vary with position.

As depicted in Fig. 8, the data showcasing changes in velocity, lateral acceleration, front wheel steering angle, and yaw rate at a speed of 50 km/h demonstrate the performance of the autonomous avoidance model equipped with the new visual algorithm. The model vehicle's velocity remains remarkably stable, staying within the vicinity of the initially set target speed. Within the framework of deep learning, the driving system efficiently detects vehicles ahead, allowing for prompt decision-making in terms of following, decelerating, and lane-changing maneuvers. It is evident that the autonomous driving system, enhanced by the new visual algorithm, possesses a stable and effective obstacle avoidance capability. This reinforces the system's ability to detect and respond to vehicles in its path, ensuring safe and reliable operation.

#### 70 km/h experiment

The system was configured with a target vehicle speed of 70 km/h, and static obstacles as well as moving vehicles were introduced into the road scenario. Additionally, the threshold for the speed of moving vehicles in the scenario was increased. This adjustment aimed to further evaluate the effectiveness of the obstacle avoidance algorithm under more challenging conditions and higher-speed scenarios. By conducting simulations with these modifications, the system's ability to successfully navigate and avoid obstacles at the increased speed threshold can be thoroughly assessed, providing valuable insights into the algorithm's performance in a variety of driving scenarios.

**Figures 9:**
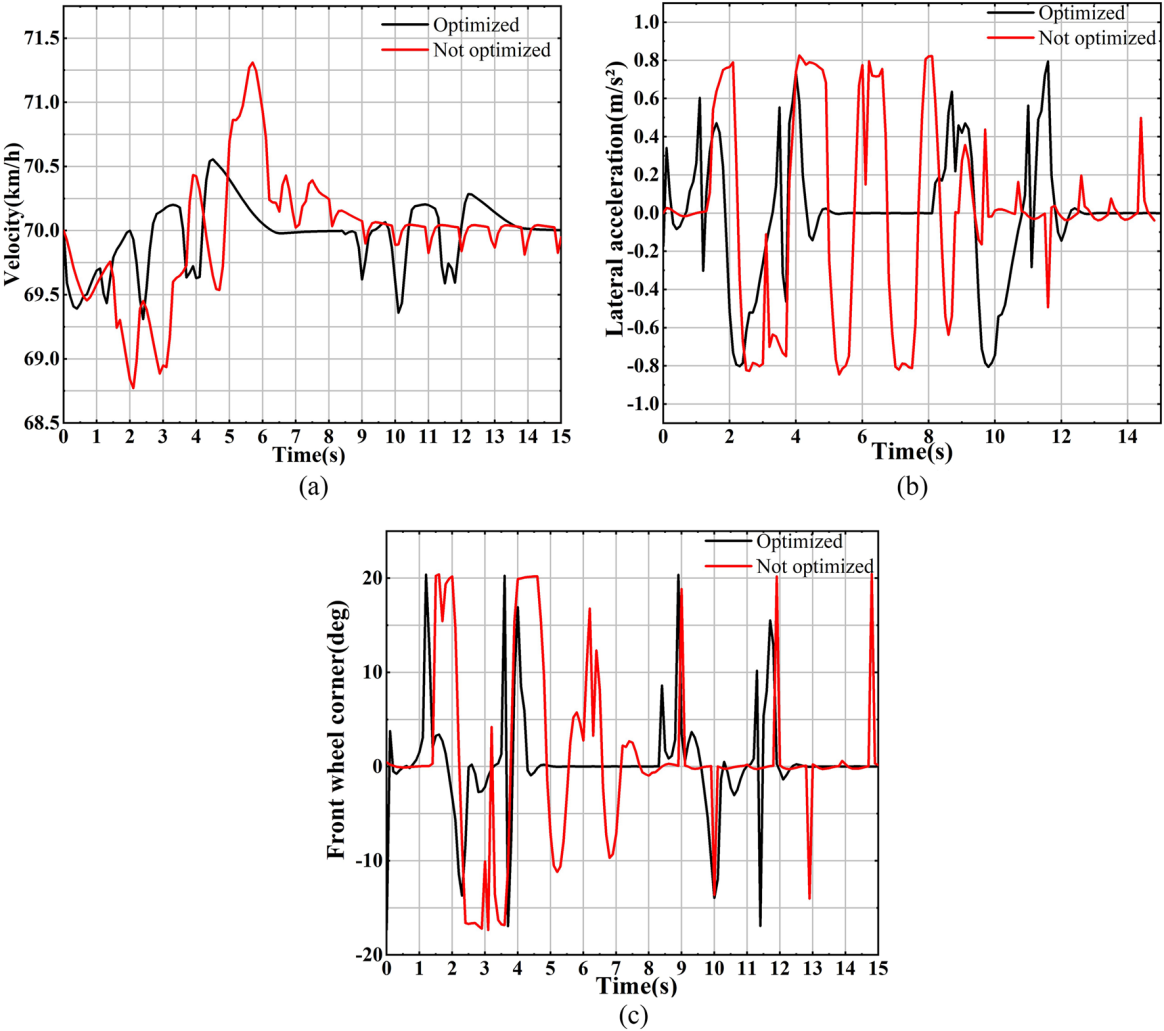
(**a**) The velocity of the vehicle before and after system optimization changes with time; (**b**) The lateral acceleration before and after system optimization changes over time; (**c**) The front wheel rotation angle before and after the system optimization changes over time.

As shown in Fig. 9a, after system optimization, the vehicle's velocity curve exhibits reduced fluctuations both in amplitude and frequency. This reduction indicates that the vehicle experiences fewer instances of acceleration and deceleration, resulting in a more stable maintenance of the target vehicle speed. Moving on to Fig. 9), the lateral acceleration curve for the optimized system demonstrates fewer fluctuations, indicating reduced occurrences of lateral deviation, with significantly lower peak values compared to the pre-optimization curve. Figure 9c further supports these improvements, indicating that the system now requires less time for steering, and there are fewer instances of large steering maneuvers.

## Results

This paper addresses the challenges faced by autonomous vehicle cameras in complex road conditions and the difficulties encountered in target identification and tracking. Leveraging the fusion of camera sensing technology and deep learning, the YOLOv5s algorithm was upgraded in three key aspects: the loss function, CBAMC3 module, and GELU activation function. During the model training process, notable improvements were observed in the convergence speed of the loss function, and the resulting model consistently achieved outstanding metrics in terms of mAP, precision, and recall. Real-world road testing of the visual model revealed that the optimized target detection algorithm efficiently and reliably acquired target position and depth information. In model testing, the optimized visual algorithm enabled the driving system to make path selections that closely approached optimal solutions. This resulted in reduced unnecessary steering and deceleration operations. The system's decision-making precision and robustness were significantly enhanced. Leveraging this exceptional visual model, the system's obstacle avoidance functionality also exhibited greater robustness. In summary, the upgrades made to the YOLOv5s algorithm, combined with rigorous real-world testing, have resulted in an optimized target detection algorithm. This algorithm enhances the system's ability to efficiently and reliably obtain target position and depth information, leading to improved decision-making precision and robustness, ultimately enhancing the system's obstacle avoidance capabilities. It can further improve traffic congestion and ensure personal safety.

## Discussion

Our research focuses solely on optical cameras. Camera vision systems detect and recognize objects by capturing and analyzing images, and are particularly adept at processing color and texture information, which is critical for understanding visual features such as traffic signs and road markings. In addition, camera devices are typically less expensive and smaller than radar systems and can be more easily integrated into existing in-vehicle systems. However, while cameras are uniquely suited to provide these important 2D image and color information, they also have limitations in self-driving vehicle applications. Camera systems are very sensitive to lighting conditions, such as nighttime or backlit environments that may greatly affect their detection capabilities.

LIDAR technology, which acquires the precise distance to an object by emitting laser pulses and measuring their reflection time, is capable of operating in light-free conditions, and its measurement accuracy is greater than that of optical cameras, although its performance may be affected by adverse weather conditions such as rain or fog^42^. This performance impact underscores the importance of employing 3D spatial data from LiDAR when modeling accurately in complex environments, especially in navigation and obstacle avoidance in self-driving vehicles. So subsequently if accuracy is to be improved then additional sensing equipment including LiDAR and radar may still be required.

Despite these challenges, the unique advantages and future potential of camera vision systems in autonomous driving cannot be ignored. Through technological innovation and interdisciplinary collaboration, camera vision will play an increasingly important role on the road to autonomous driving. In response to the limitations of camera vision systems, future research is likely to focus on improving these limitations using advanced algorithms, such as using deep learning to improve detection under unfavorable lighting conditions or enhancing depth perception through sensor fusion techniques. In addition, a combination of complementary e.g. LIDAR and radar sensing devices may still be required for optimal environmental sensing.

## Data Availability

The collision avoidance experiment datasets generated during the current study are available in the Mendeley Data repository, https://data.mendeley.com/datasets/f2kkskc55s/1. The self-made datasets are available from the corresponding author on reasonable request.

## List of symbols

{f_i_} {g_i_}: channel function
$\tilde{M}$: Augmentation matrix of M
$\tilde{m}$: Augmentation matrix of m
s: Measurement factor
A: Internal reference matrix
Ri: Rotation matrix
Ti: Pan vectors
r: Matrix parameters
λ: Matrix parameters
α: Matrix parameters
β: Matrix parameters
γ: Matrix parameters
B: Symmetry matrix
k: Distortion coefficient
m_ij_: Figure i Point j coordinates
v: Matrix parameters
(u_0_,v_0_): Camera point coordinates
D: Matrix of coefficients
d: The difference in pixel coordinates without distortion
$\hat{m}$: The coordinates after calculating the distortion distortion
l_box_: Loss of location
l_obj_: Confidence loss
l_cls_: Classified losses
(x_p_, y_p_): Predict box coordinates
(x_t_, y_t_): Prediction box’s width
w_p_: Prediction box’s width
h_p_: Prediction box’s height
w_t_: Target box’s width
h_t_: Prediction box’s height

## Abbreviations

YOLO: You only look once
GELU: Gaussian error linear unit
MPC: Model predictive control
ADAS: Advanced driver assistance systems
CNN: Convolutional neural network
RELU: Rectified linear unit
CBAM: Convolutional block attention module
SSD: Single shot multiBox detector
SLAM: Simultaneous localization and mapping
LSTM: Long short-term memory
CSP: Cross-stage partial networks
LSTM-FCN: Long short-term memory fully convolutional network
SPP: Spatial pyramid pooling
mAP: mean average precision
